# Activation of transient receptor potential vanilloid 4 involves in hypoxia/reoxygenation injury in cardiomyocytes

**DOI:** 10.1038/cddis.2017.227

**Published:** 2017-05-25

**Authors:** Qiong-Feng Wu, Cheng Qian, Ning Zhao, Qian Dong, Jing Li, Bin-Bin Wang, Lei Chen, Lixiu Yu, Bing Han, Yi-Mei Du, Yu-Hua Liao

**Affiliations:** 1 Department of Cardiology, Union Hospital, Tongji Medical College, Huazhong University of Science and Technology, Wuhan, China; 2 Research Center of Ion Channelopathy, Union Hospital, Tongji Medical College, Huazhong University of Science and Technology, Wuhan, China; 3 Institute of Cardiology, Union Hospital, Tongji Medical College, Huazhong University of Science and Technology, Wuhan, China; 4 Key Lab for Biological Targeted Therapy of Education Ministry and Hubei Province, Union Hospital, Tongji Medical College, Huazhong University of Science and Technology, Wuhan, China; 5 Department of Physiology, Nanjing Medical University, Nanjing, China; 6 Department of Pharmacy, Union Hospital, Tongji Medical College, Huazhong University of Science and Technology, Wuhan, China; 7 Department of Cardiology, Xuzhou Central Hospital, Xuzhou, China

## Abstract

Transient receptor potential vanilloid 4 (TRPV4) is highly expressed in heart and vessels and can be activated during myocardial ischemia/reperfusion (I/R). Recently, we found that treatment with a selective TRPV4 antagonist HC-067047 significantly reduced infarct size, decreased troponin T levels and improved cardiac function in murine model myocardial I/R. This study was undertaken to investigate the mechanism underlying TRPV4-mediated myocardial I/R injury. To mimic myocardial I/R injury, we established a hypoxia/reoxygenation (H/R) model in H9C2 cells and neonatal rat ventricle myocytes (NRVMs) *in vitro*. TRPV4 mRNA and protein expression was confirmed in the H9C2 and NRVM, whereas functional TRPV4 activity was assessed from Ca^2+^ influx response to a TRPV4 agonist GSK1016790A. TRPV4 functional expression was significantly enhanced during H/R. Furthermore, H/R increased the intracellular Ca^2+^ concentration ([Ca^2+^]_i_) and induced cell injury, which were reversed by HC-067047 but was further aggravated by GSK1016790A. Moreover, HC-067047 treatment significantly alleviated the increase of reactive oxygen species (ROS) generation, the depolarization of mitochondrial membrane potential (Δψm) and the opening of mitochondrial permeability transition pore (mPTP) during H/R. On the contrary, GSK1016790A exacerbated those effects. Meanwhile, increase in [Ca^2+^]_i_ and ROS induced by activation of TRPV4 was almost abolished when cells were cultured in Ca^2+^-free medium. In addition, ROS scavenger NAC obviously reversed activation of TRPV4-induced changes of Δψm and mPTP opening. Finally, we confirmed the direct roles of TRPV4 on cardiac injury and ROS generation in murine model myocardial I/R *in vivo*. In conclusion, activation of TRPV4 induces Ca^2+^ influx in cardiomyocytes, with subsequent ROS release, depolarizing of Δψm, opening mPTP, inducing injury and TRPV4 has key roles during I/R via these pathways.

TRPV4 belongs to the transient receptor potential vanilloid (TRPV) subfamily of transient receptor potential (TRP) cation channels. It is widely distributed in various organs and tissues including heart and vessels.^1, 2^ TRPV4 may function as a molecular integrator of multiple chemical and physical stimuli, including hypotonic stimulation, cell swelling, moderate heat (>24–37 °C), endogenous metabolites of arachidonic acid and synthetic chemical ligands (such as GSK1016790A and 4*α*-PDD).^3^ Therefore, TRPV4 may have an important role in modulating the function of the cardiovascular system in physiological and pathological conditions.^4^

During myocardial ischemia/reperfusion (I/R), TRPV4 may be overactivated by cytotoxic edema or the metabolites of arachidonic acid, and it thus involves in myocardial I/R injury.^5, 6^ Indeed, our previous study has demonstrated that treatment with a selective TRPV4 antagonist HC-067047 significantly reduced infarct size, decreased troponin T levels and improved cardiac function in murine model myocardial I/R.^7^ Increased activation of TRPV4 has been also observed in the hippocampus after cerebral I/R, whereas blocking TRPV4 attenuated I/R-induced brain injury.^8, 9, 10, 11^ Furthermore, sustained activation of TRPV4 dose-dependently induces apoptosis of retinal ganglion cells and neuronal death in the hippocampus.^12, 13^ Therefore, TRPV4 may be a promising target to prevent myocardial I/R injury, but the mechanism underlying TRPV4-mediated myocardial I/R injury is still unclear.

TRPV4 is highly permeable to Ca^2+^. Activation of TRPV4 elicits Ca^2+^ influx and increases the intracellular concentration of free Ca^2+^ ([Ca^2+^]_i_).^1, 14^ Furthermore, recent studies have found that TRPV4 agonists enhances the production of reactive oxygen species (ROS) via Ca^2+^ influx in endothelial cells, urothelial cell, macrophages, as well as hippocampus.^15, 16, 17, 18^ In addition, Ca^2+^ overload and high level of ROS can trigger depolarization of mitochondrial membrane potential (Δψm), opening of the mitochondrial permeability transition pore (mPTP) and result in cell death, which contribute to myocardial I/R injury.^19^ Therefore, we hypothesized that TRPV4 activation exacerbates myocardial I/R injury via Ca^2+^/ROS/mPTP pathway. To mimic myocardial I/R injury, we established a hypoxia/reoxygenation (H/R) model in H9C2 cells and neonatal rat ventricle myocytes (NRVMs) *in vitro*. Some of the results were confirmed in the murine model myocardial I/R *in vivo*.

## Results

### TRPV4 is functional expressed in cardiomyocytes

**Figure 1:**
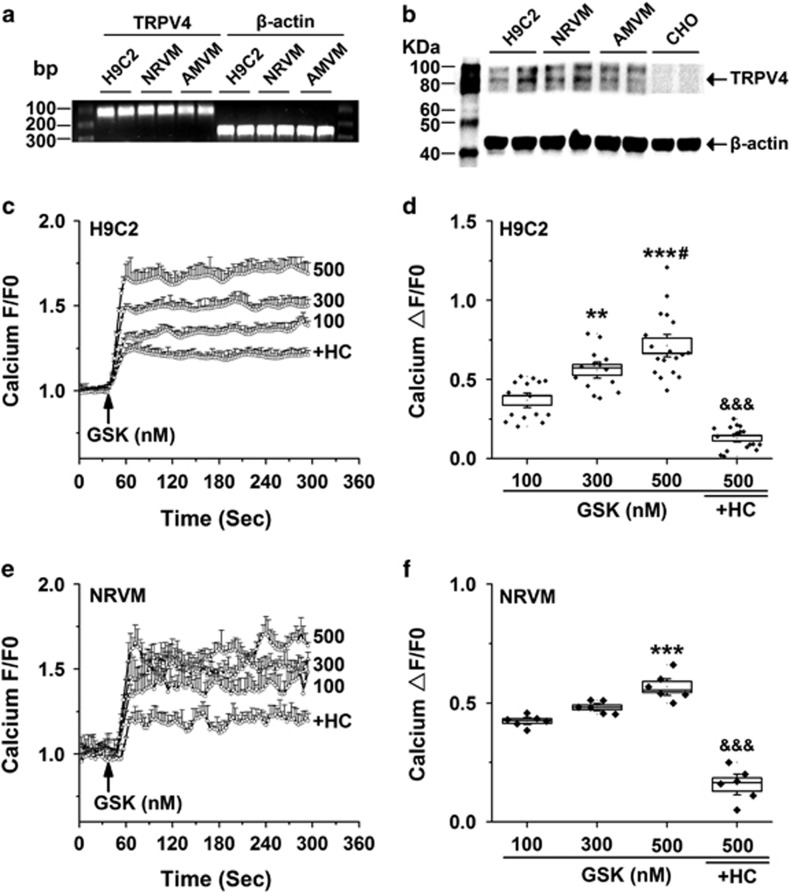
The functional expression of TRPV4 channel in cardiomyocytes. RT-PCR (**a**) and western blot analysis (**b**) showing the expression of TRPV4 mRNA and protein in H9C2, NRVM, AMVM and CHO. Representative graphs showing that the changes in [Ca^2+^]_i_ levels in H9C2 (**c**) and NRVM (**e**) in response to the TRPV4 agonist GSK1016790A (100, 300 and 500 nM). Arrow indicates the addition of the stimulator. In some experiments, cells were pretreated with 1 *μ*M HC-067047 for 30 min. Quantitative analysis of relative changes (ΔF/F0) in Ca^2+^ influx induced by GSK1016790A in H9C2 (**d**) and NRVM (**f**) loaded with Fluo-4/AM. The data are represented as mean±S.E.M. from ≥6 independent experiments. **P<0.01, ***P<0.001 *versus* GSK at 100 nM; ^#^*P*<0.05 *versus* GSK at 300 nM, ^&&&^*P*<0.001 *versus* GSK at 500 nM

We first determine whether TRPV4 is expressed in cardiomyocytes using RT-PCR and western blot techniques. As shown in Figure 1a, TRPV4 mRNA was detected positively at 108 bp in H9C2, NRVM and adult mice ventricle myocytes (AMVMs). Correspondingly, TRPV4 protein was presented with two obvious bands of molecular weights about 85 and 100 kDa as expected, but absent in Chinese hamster ovary (CHO) cells (Figure 1b). To further test whether TRPV4 is functionally active in cardiomyocytes, we loaded H9C2 and NRVM with Fluo-4/AM and measured Ca^2+^ influx in response to the specific TRPV4 agonist, GSK1016790A. GSK1016790A induced robust concentration-dependent (100, 300 and 500 nM) Ca^2+^ influx in H9C2, which was almost blocked by pretreatment with a selective TRPV4 antagonist, HC-067047 (1 *μ*M) for 30 min (Figure 1c). Summary data were shown in Figure 1d. A similar Ca^2+^ response to GSK1016790A and HC-067047 was observed in NRVM (Figures 1e and f). Taken together, our results show that TRPV4 is functionally expressed in cardiomyocytes.

### The functional expression of TRPV4 is upregulated in cardiomyocytes subjected to H/R

**Figure 2:**
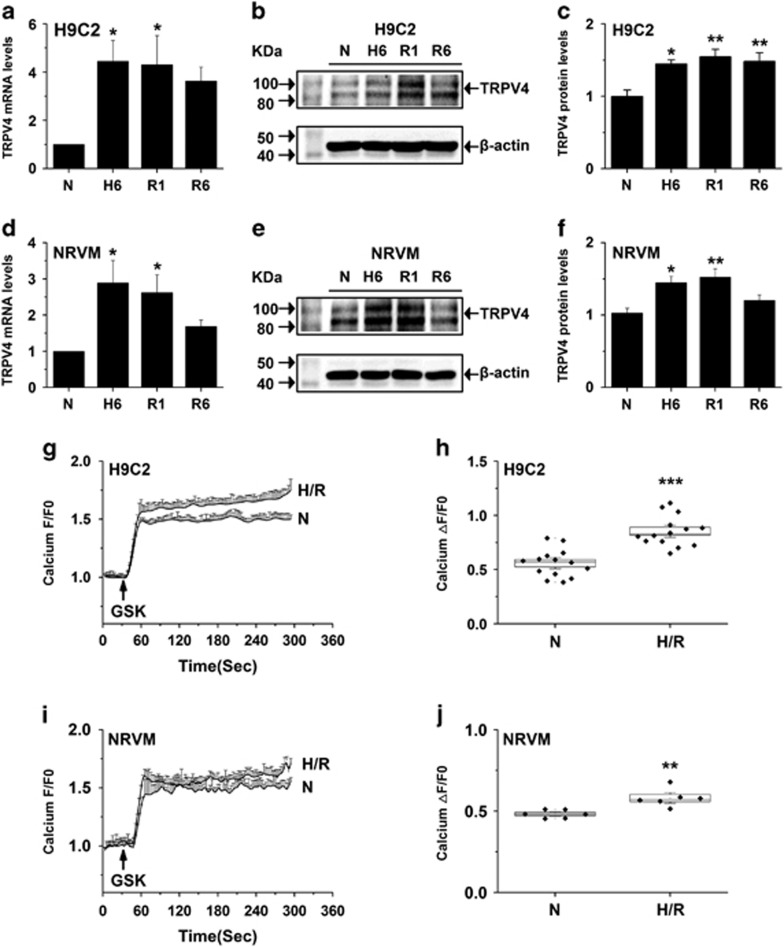
Upregulated functional expression of TRPV4 in cardiomyocytes after exposed to H/R. Real-time PCR analysis of TRPV4 mRNA in H9C2 (**a**) and at NRVM (**d**) at different times after H/R. Western blot analysis of TRPV4 protein in H9C2 (**b** and **c**) and at NRVM (**e** and **f**) at different times after H/R. Representative graphs showing that Ca^2+^ influx induced by GSK1016790A (300 nM) in H9C2 (**g**) and NRVM (**i**). Summary data for maximal increases in Ca^2+^ influx induced by GSK1016790A in H9C2 (**h**) and NRVM (**j**). The results shown are mean±S.E.M. from ≥6 independent experiments. *P<0.05, **P<0.01,***P<0.001 *versus* N

As shown in Figures 2a and c, TRPV4 mRNA and protein expression levels markedly increased after 6-h hypoxia and maintained higher level at 1-h reoxygenation, but began to decrease at 6-h reoxygenation in H9C2. Similar results were observed in NRVM (Figures 2d and f). Same as previous observations,^8, 12^ we found H/R obviously increased the Ca^2+^ influx responses to 300 nM GSK1016790A in H9C2 (Figure 2g) and NRVM (Figure 2i). Figures 2h and j show the quantitative analysis of relative changes (ΔF/F0) in Ca^2+^ influx at the steady state in H9C2 and NRVM cells, respectively. Our results indicate that H/R increases the functional expression of TRPV4.

### Activation of TRPV4 contributes to Ca^2+^ overload in cardiomyocytes subjected to H/R

**Figure 3:**
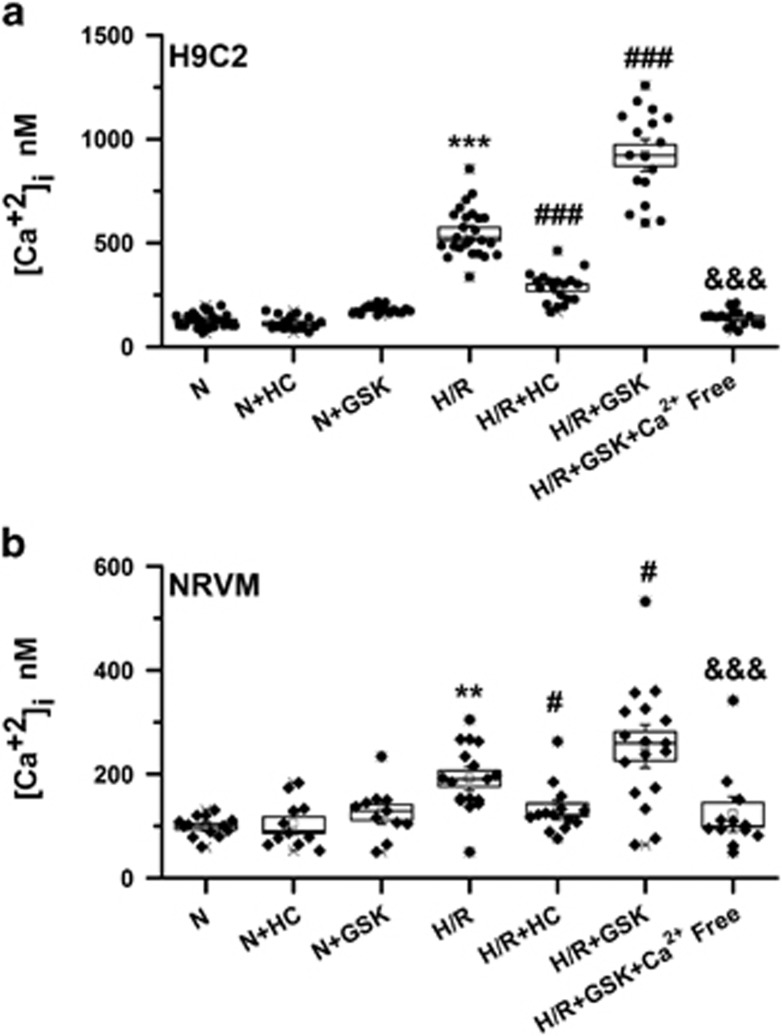
Effects of TRPV4 antagonist HC-067047 and TRPV4 agonist GSK1016790A on [Ca^2+^]_i_ in H9C2 and NRVM subjected to H/R. [Ca^2+^]_i_ was detected by fluorescent fluo-4/AM staining in H9C2 (**a**) and NRVM (**b**). Cultured cells were exposed to H/R and incubated with HC-067047 (1 *μ*M) or GSK1016790A (300 nM) during reoxygenation. The results are represented as mean±S.E.M. from ≥10 independent experiments, **P<0.01, ***P<0.001 *versus* N; ^#^*P*<0.05 ^###^*P*<0.001 *versus* H/R; ^&&&^*P*<0.001 *versus* H/R+GSK

Consistent with previous reports,^20, 21^ H/R-induced [Ca^2+^]_i_ overload (550.51±23.34 nM *versus* 178.44±4.60 nM, *P*<0.001 *versus* N), which can be reduced and enhanced by HC-067047 (285.91±17.02 nM, *P*<0.001 *versus* H/R) and GSK1016790A (922.59±51.43 nM, *P*<0.001 *versus* H/R) in H9C2 (Figure 3a), respectively. Similarly, [Ca^2+^]_i_ was obviously elevated in NRVM (Figure 3b) subjected to H/R (193.72 ±16.08 nM *versus* 97.44 ±5.59 nM, *P*<0.01 *versus* N), which was significantly blunted in the presence of HC-067047 (120.87±7.83 nM, *P*<0.05 *versus* H/R) and aggravated by GSK1016790 (264.54±27.10 nM, *P*<0.05 *versus* H/R). Importantly, the increase [Ca^2+^]_i_ induced by GSK1016790 during H/R was completely abolished or strongly reduced when cells were cultured in Ca^2+^-free medium, consistent with the concept that the [Ca^2+^]_i_ induced by activation of TRPV4 was mainly from extracellular Ca^2+^ influx. Interestingly, HC-067047 and GSK1016790A did not influence [Ca^2+^]_i_ under normoxic conditions (Figures 3a and b). Our results suggest that activation of TRPV4 involves in H/R-induced [Ca^2+^]_i_ overload in the cardiomyocytes.

### Activation of TRPV4 involves in H/R-induced injury in cardiomyocytes

**Figure 4:**
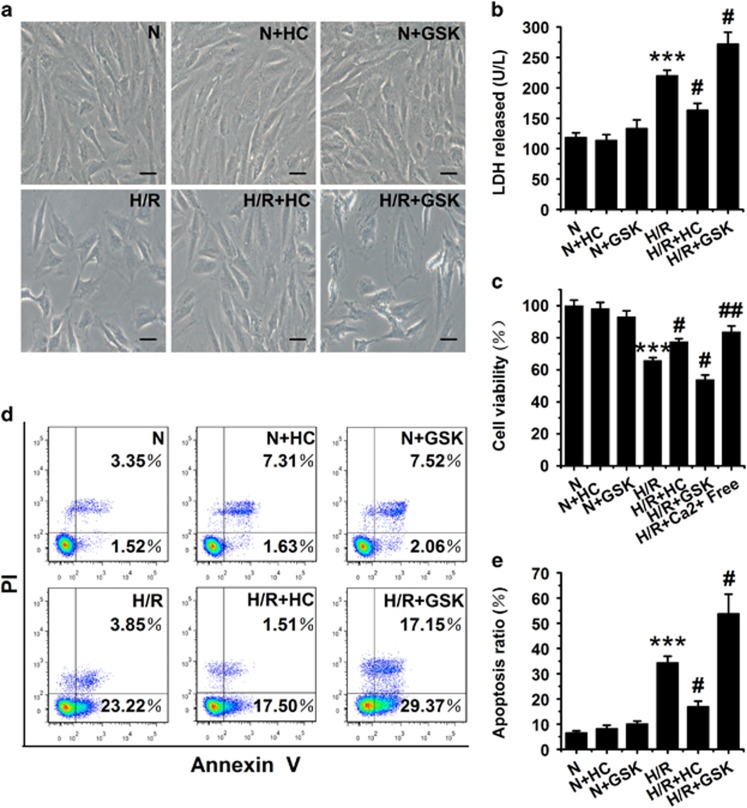
Effects of TRPV4 antagonist HC-067047 and TRPV4 agonist GSK1016790A on H/R-induced cell morphology change, LDH release, cell viability and apoptosis in H9C2 subjected to H/R. (**a**) Light microscopic images of morphological changes. Scar bar: 25 *μ*m. (**b**) LDH release was measured by LDH assay kits. (**c**) Cell viability was detected using CCK-8 assay. Representative images (**d**) and quantitative analysis (**e**) of apoptotic rate was assessed as (Annexin V (+) PI (-) cells+ Annexin V (+) PI (+) cells)/total cells × 100% using flow cytometry. Cultured cells were exposed to H/R and incubated with HC-067047 (1 *μ*M) or GSK1016790A (300 nM) during reoxygenation. The results are represented as mean±S.E.M. from ≥6 independent experiments. ***P<0.001 *versus* N, ^#^*P*<0.05 *versus* H/R

To test whether activation of TRPV4 involved in H/R-induced injury in cardiomyocytes, we investigated the effects of inhibition or activation of TRPV4 on the H/R-induced change of cell morphology (Figure 4a), lactate dehydrogenase (LDH) release (Figure 4b), cell viability (Figure 4c) and cell apoptosis (Figures 4d and e) in H9C2 cells. In accord with previous reports, H/R-induced significant injuries in H9C2 cells. Inhibition of TRPV4 by 1 *μ*M HC-067047 during reoxygenation obviously reduced the H/R-induced injury, whereas activation of TRPV4 by 300 nM GSK1016790A aggravated H/R-induced injury. Meanwhile, either HC-067047 or GSK1016790A did not show any obvious effects on cell morphology, LDH release, cell viability and cell apoptosis under normoxic condition. Interestingly, cell viability has obviously recovered when the cells were cultured in Ca^2+^-free medium during H/R.

### TRPV4-mediated Ca^2+^ influx involves in cytoplasmic ROS generation following H/R

**Figure 5:**
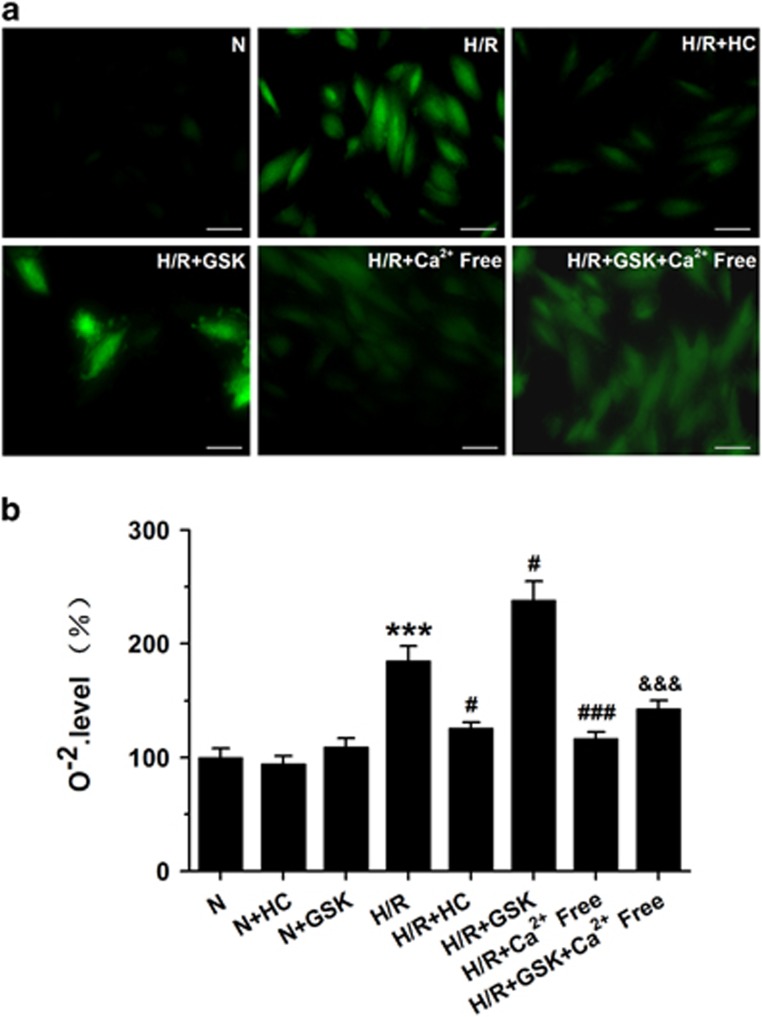
Effects of TRPV4 antagonist HC-067047 and TRPV4 agonist GSK1016790A on ROS generation in H9C2 subjected to H/R. Representative images (**a**) and quantitative analysis (**b**) of ROS generation was assessed by DCFH-DA labeling using fluorescence microscopy and a Enspire multimode plate reader, respectively. Scar bar: 50 *μ*m. The results are represented as mean±S.E.M. from ≥6 independent experiments, ***P<0.01 *versus* N; ^#^*P*<0.05 ^###^*P*<0.001 *versus* H/R; ^&&&^*P*<0.001 *versus* H/R+GSK

To test the hypothesis that TRPV4 activation may enhance oxidative injury during H/R, we quantified O^2-^ content using DHE staining. Original fluorescent images and the quantitative analysis have been shown in Figures 5a and b, respectively. As expected, blockade TRPV4 by HC-067047 significantly attenuated H/R-induced increase in O^2-^ formation, whereas activation TRPV4 with GSK1016790A treatment further increased this effect. However, the enhancement effects on ROS generation in both in H/R and H/R+GSK1016790A groups were almost abolished when the cells were cultured in Ca^2+^-free medium, suggesting that Ca^2+^ influx mediated by TRPV4 activation involves in the ROS generation during H/R. Either HC-067047 or GSK1016790A has no effects on ROS generation under normoxic conditions (Supplementary Figure S1).

### ROS generation mediated by TRPV4 activation induces mPTP opening in cardiomyocytes subjected to H/R

**Figure 6:**
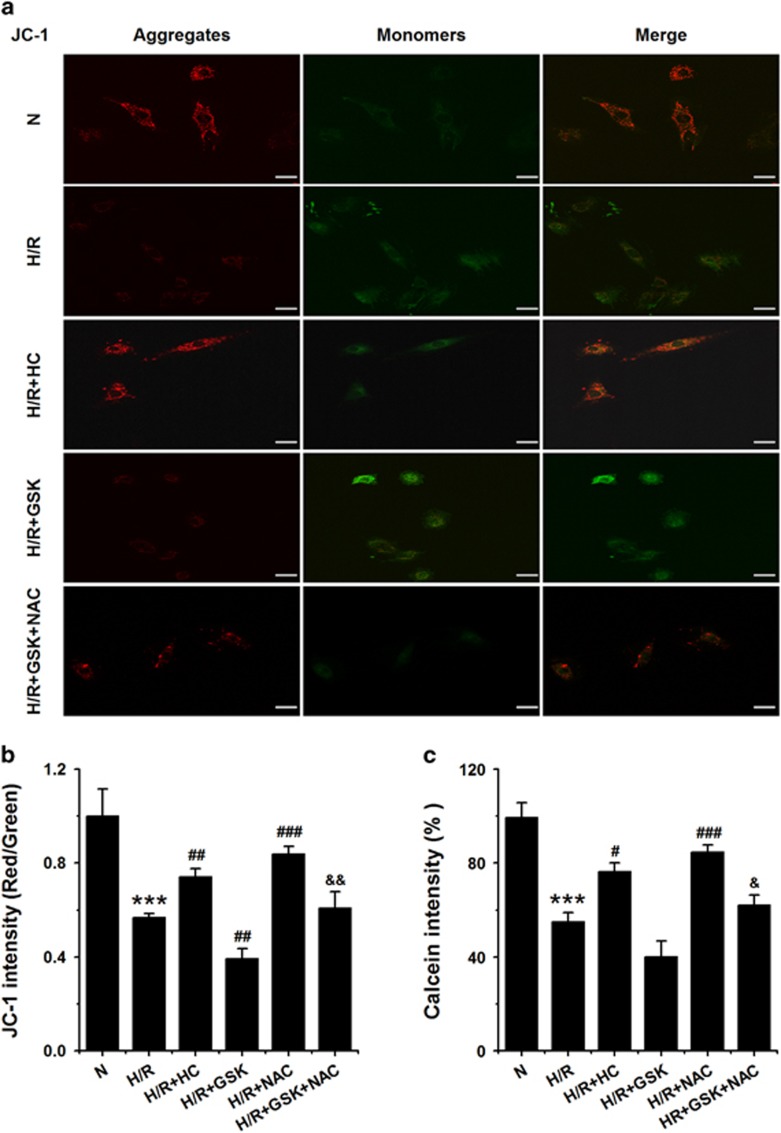
Effects of TRPV4 antagonist HC-067047 and TRPV4 agonist GSK1016790A on ΔΨm and mPTP opening levels in H9C2 subjected to H/R. Representative images (**a**) and quantitative analysis (**b**) of ΔΨm level was assessed by the lipophilic cationic probe JC-1 using fluorescence microscopy and a Enspire multimode plate reader, respectively. Scar bar: 50 *μ*m (**c**) mPTP opening was measured with the ratio of calcein fluorescences density using a Enspire multimode plate reader. The results are represented as mean±S.E.M. from ≥6 independent experiments, ***P<0.001 *versus* N; ^#^*P*<0.05, ##*P*<0.01, ^###^*P*<0.001 *versus* H/R; ^&^*P*<0.05, ^&&^*P*<0.001 *versus* H/R+GSK

We next sought to investigate downstream effectors of ROS that mediate TRPV4 activation induced injury in H9C2 cells subjected to H/R. It has been shown that ROS involves in H/R-induced injury via effects on the depolarization of ΔΨm and the opening of mPTP.^22^ Δψm was measured using JC-1 staining and calculated as the fluorescent ratio of red to green. The lower ratio illustrated the level of mitochondrial depolarization. As shown in Figures 6a and b, H/R-induced depolarization of ΔΨm, which was partially reversed by HC-067047 treatment but further enhanced by GSK1016790A. Moreover, we detected the mPTP opening mode with the calcein-cobalt method. Consistent with the above results, HC-067047 blocked the H/R-induced mPTP opening, whereas GSK1016790A enhanced the H/R-induced mPTP opening (Figure 6c). As expected, 8 mM NAC significantly restored ΔΨm and inhibition of mPTP opening during H/R and H/R+GSK1016790A (Figure 6). Thus, mPTP is an important downstream effector in the activation of TRPV4-ROS induced cardiotoxicity during H/R.

### Activation of TRPV4 involves myocardial I/R injury *in vivo*

**Figure 7:**
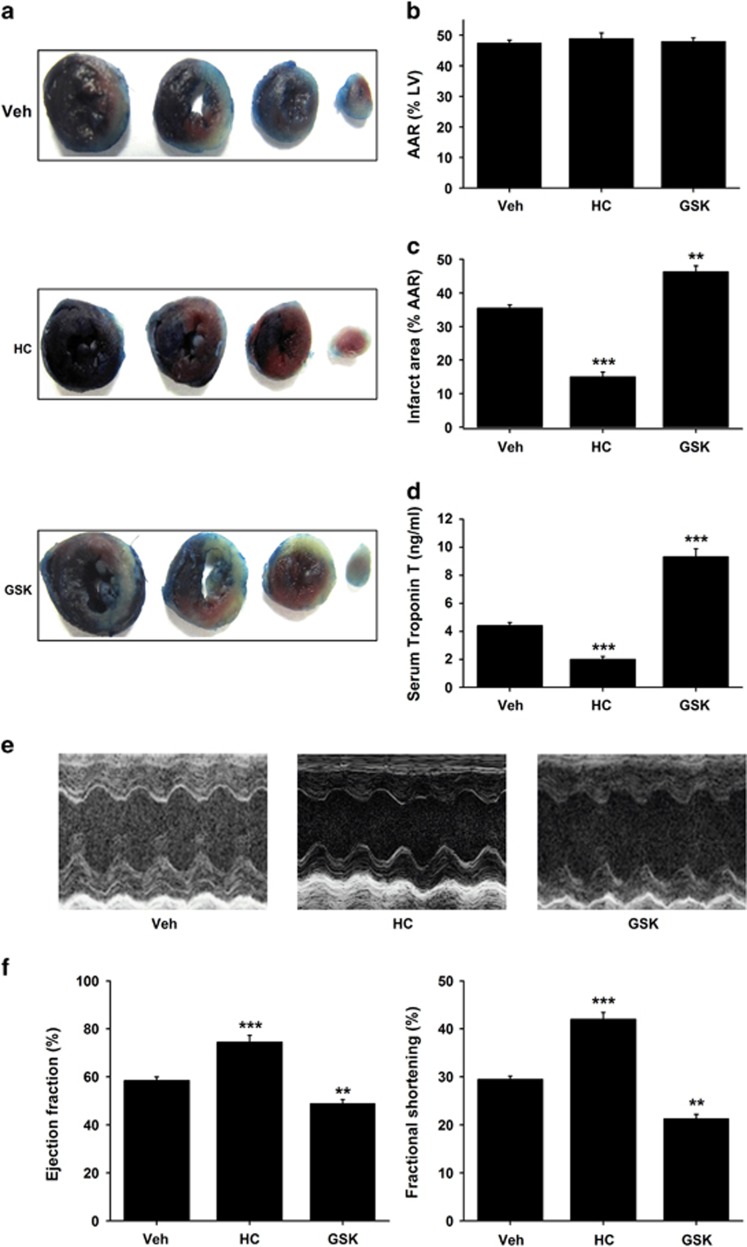
Effects of TRPV4 antagonist HC-067047 and TRPV4 agonist GSK1016790A on infarct size, serum cardiac troponin T and heart function in a mice model of myocardial I/R. (**a**) Representative images of LV slices from different groups at 24 h after reperfusion as stained by Evan’s Blue and TTC. The non-ischemic area is indicated in blue, the AAR in red, and the IA in white. (**b**) Quantification of AAR (%LV) at 24 h after reperfusion. (**c**) Quantification of infarct size of myocardial tissues at 24 h after reperfusion. (**d**) Serum concentration of TnT at 24 h after reperfusion. (**e**) Representative M-mode echocardiography images of the LV at 24 h after reperfusion. (**f**) Quantification of LV EF and FS. The TRPV4 antagonist HC-067047 (10 mg/kg) was intraperitoneally injected beginning at 1 h after reperfusion and then injected every 8 h, and the TRPV4 agonist GSK1016790A (0.025 mg/kg) were administrated via jugular vein on the onset of reperfusion. The results are represented as mean±S.E.M., *n*=6 per group, **P<0.01,***P<0.001 *versus* Vehicle

We further investigated the effects of inhibition or activation of TRPV4 on infarct size, serum cardiac troponin T (cTnT) level and heart function in myocardial I/R injury *in vivo*. Figure 7a showed representative photographs of heart tissues stained with Evans blue dye to delineate the area at risk (AAR) and TTC to delineate the infarct area (IA). Mice treated with HC-067047 showed a significantly reduced infarct size by 58% compared with vehicle (15±1.4% *versus* 36±1%, *P*<0.001; Figure 7c). In contrast, GSK1016790A group, the myocardial infarct size was greater compared with vehicle group (46±1.76%, *P*<0.01; Figure 7c). AAR was similar among groups (Figure 7b). Furthermore, serum cardiac troponin T level, a marker of cardiac injury, was significantly lower in the HC-067047 group, but higher in the GSK1016790A group (Figure 7d). Consistent with infarct size, a marked improved of cardiac function, as shown by increases in ejection fraction (EF) and fractional shortening (FS) measured at 24 h after reperfusion, was observed in the HC-067047 group (Figures 7d and f). On the contrary, the EF and FS was remarkably reduced in GSK1016790A groups compared with vehicle group (Figures 7e and f). Our results confirm the direct role of TRPV4 during myocardial I/R *in vivo*.

### Activation of TRPV4 involves ROS generation during myocardial I/R *in vivo*

**Figure 8:**
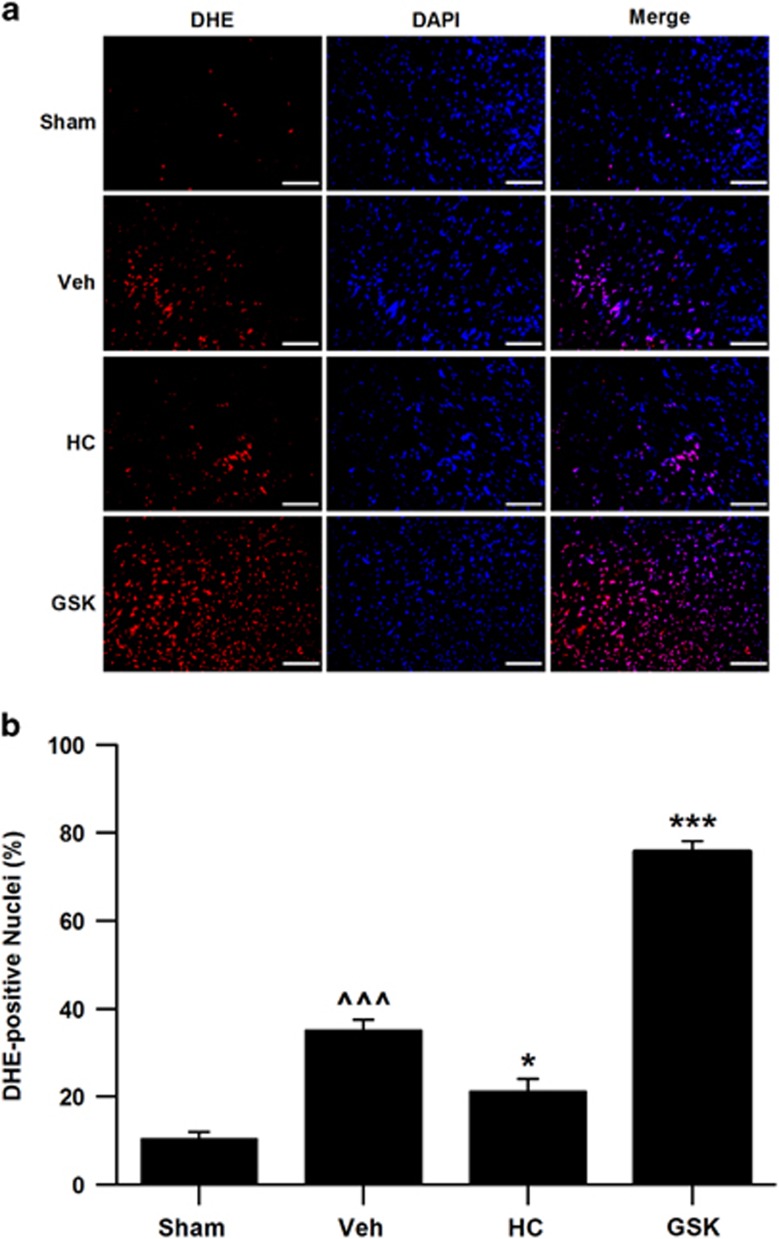
Effects of TRPV4 antagonist HC-067047 and TRPV4 agonist GSK1016790A on the cardiac oxidative stress in a mice model of myocardial I/R. (**a**) ROS production in the LV sections was evaluated with dihydroethidium (DHE) staining at 4 h after reperfusion. ROS-positive cells were identified by DHE staining (red), and total nuclei by DAPI staining (blue). Scale bar: 100 *μ*m. (**b**) Quantitative analysis of ROS, as normalized to sham. The TRPV4 antagonist HC-067047 (10 mg/kg) was intraperitoneally injected beginning at 1 h after reperfusion and the TRPV4 agonist GSK1016790A (0.025 mg/kg) were administrated via jugular vein on the onset of reperfusion. *n*=6–8 per group, ^^^*P*<0.001 *versus* Sham, *P<0.05, ***P<0.001 *versus* Vehicle

We also measured the effects of inhibition or activation of TRPV4 on ROS generation in myocardial I/R injury *in vivo* (Figure 8). This results show that myocardial I/R significantly increased ROS levels, which was reduced by treatment with HC-076047 but was enhanced by GSK1016790A. This was consistent with the results from the H9C2 cells *in vitro*.

## Discussion

TRPV4 is widely expressed in the cardiovascular system with functional existence in endothelial cells, smooth muscle cells and cardiac fibroblasts.^23, 24, 25, 26, 27^ Activation of TRPV4 has been found to be involved in cardiac remodeling, pulmonary hypertension, blood pressure regulation and congestive heart failure.^28, 29, 30, 31, 32^ Our previous study has highlighted that TRPV4 have important roles in myocardial I/R-induced injury.^7^ In this study, we explored the mechanism underlying TRPV4-mediated myocardial I/R injury. Our results have shown that activation of TRPV4 induces Ca^2+^ influx in cardiomyocytes, with subsequent ROS release, depolarizing of Δψm, opening mPTP, inducing injury and TRPV4 has key roles during myocardial I/R via these pathways.

Consistent with our previous observation *in vivo* myocardial I/R, we showed the levels of TRPV4 mRNA and protein increased in cultured cardiomyocytes during H/R. Correspondingly, a greater Ca^2+^ influx induced by TRPV4 agonist GSK1016790A was observed in cultured cardiomyocytes after being exposed to H/R, indicating that the TRPV4 functional activity is enhanced during H/R. This increase in Ca^2+^ entry could have important functional consequences. Ca^2+^ entry mediated via TRPV4 has been reported to trigger apoptosis in several cells. For example, application of TRPV4 agonist promoted the dose-dependent apoptosis of retinal ganglion cells and neurons in the hippocampus.^12, 13^ A similar finding of the increase TRPV4 expression and TRPV4-mediated Ca^2+^ entry has been described in neuronal injury after cerebral I/R.^8, 9^ Therefore, we hypothesize that activation of TRPV4 involved in myocardial I/R-induced injury.

Although a number of Ca^2+^ entry pathways have been implicated in mediating cardiomyocyte Ca^2+^ overload following myocardial I/R, including the Na^+^/Ca^2+^ exchanger and the L-type Ca^2+^ channel, there is still considerable controversy as to which pathways are critical in mediating this process.^33, 34, 35^ Elevated [Ca^2+^]_i_ is believed to be a central mediator of myocardial injury.^35^ In agreement with previous studies, we found that [Ca^2+^]_i_ was increased in cultured H9C2 and CMs after exposed H/R. We have also shown that TRPV4 antagonist HC-067047 markedly ameliorated H/R-induced injury *in vitro* as well as I/R-induced injury *in vivo*, consistent with attenuation of H/R-induced increase in [Ca^2+^]_i_. On the contrary, application TRPV4 agonist GSK1016790A exacerbated H/R or I/R-induced injury, and meanwhile, accompanying additional increase in [Ca^2+^]_i_. Moreover, increase in [Ca^2+^]_i_ induced by activation of TRPV4 during H/R was almost abolished when cells were cultured in Ca^2+^-free medium. These results suggest that Ca^2+^ entry via TRPV4 may contribute to Ca^2+^ overload and injury following myocardial I/R.

Increase in ROS is also a key mediator of myocardial I/R injury.^19, 22^ In this study, we found that TRPV4 antagonist HC-067047 attenuated ROS increase in H9C2 cells during H/R, however, TRPV4 agonist GSK1016790A treatment induced further increase, suggesting that TRPV4 may be involved in ROS production. In addition, activation TRPV4 induced ROS generation were almost abolished when cells were cultured in Ca^2+^-free medium, indicating that Ca^2+^ influx via TRPV4 seems the mainly contributor of ROS generation. Indeed, Ca^2+^ entry via TRPV4 has been shown to increase mitochondrial ROS production in many different cells.^15, 16, 17, 18^ Activation of TRPV4 contributing to ROS production also confirmed in myocardial I/R injury.

Excessive ROS generation triggers the depolarization of ΔΨm and the opening of mPTP, which initiates death pathways. Correspondingly, treatment the cells with HC-067047 and GSK1016790A during reoxygenation significantly alleviated and accelerated Δψm depolarization and mPTP opening, respectively. Meanwhile, ROS scavenger NAC obviously reversed TRPV4 activation induced Δψm loss and mPTP opening during H/R. Thus, ROS are involved activation of TRPV4 induced Δψm loss and mPTP opening during H/R.

In summary, our results show that Ca^2+^ entry via TRPV4 may involve in ROS production and then induce the depolarization of ΔΨm and the opening of mPTP, finally lead to cell death and apoptosis during myocardial I/R injury. Our findings provide a novel cellular mechanism involved in the pathophysiology of myocardial I/R injury.

## Materials and methods

### Cell isolation and culture

Both rat heart tissue-derived H9C2 cardiac myoblast cell line and CHO cell line from ATCC (Rockefeller, MD, USA) were cultured in Dulbecco's modified Eagle's medium (DMEM, Gibco, Grand Island, NY, USA, #12800), supplemented with 15% fetal bovine serum (FBS, Hangzhou Sijiqing Biological Engineering Materials Co., Ltd., Hangzhou, China) at 37 °C in a humidified atmosphere of 95% air and 5% CO_2_. NRVM were isolated from the whole heart of 1- to 2-day-old Sprague–Dawley rats using a modification of a previously described protocol.^36, 37^ Briefly, hearts were minced, digested with enzymatic solution containing 0.1% (type II, Worthington Biochemical, Lakewood, NJ, USA) and 0.25% trypsin 1:250 (Amresco, Fountain Parkway Solon, OH, USA, #0458) for 8 consecutive 7–10 min treatment periods at 37 °C. Cells were washed and then resuspended in 4.5 g/l glucose DMEM supplemented with FBS, and 1% penicillin/streptomycin (v/v) and 100 *μ*M bromodeoxyuridine (Sigma, St. Louis, MO, USA #858811) was added. AMVM were isolated from the heart of 8–12 weeks adult C57BL/6 mice using a langendorff apparatus. After the animals were anesthetized with ether, their hearts were removed and retrograde perfusion through the aorta by 1 mg/ml collagenase type II for 10–15 min. H9C2 and NRVM were treated with a TRPV4 selective agonist GSK1016790A (Sigma-Aldrich, St. Louis, MO, USA) and a TRPV4 selective antagonist HC-067047 (Sigma-Aldrich, St. Louis, MO, USA) at the onset of reoxygenation.

### H/R model

When NRVM and H9C2 cells reached 80% confluence, hypoxia was induced by replacing the air content with a 95% N_2_ and 5% CO_2_ gas mixture in a controlled hypoxic plastic chamber (HiTech Photelectricity Biotechnology Co., Ltd, Guangzhou, China) and replacing the media with fresh l.5 g/l glucose DMEM (DMEM, Gibco, Grand Island, NY, USA) without serum for 6 h. Subsequently, the medium was replaced by the normal media and incubated in 95% air and 5% CO_2_ for 6- h reoxygenation. Normal groups were cultured in normoxic conditions for corresponding times.

### Reverse transcription (RT)-PCR amplification and quantitative (q) PCR

Total RNAs were extracted from cultured H9C2, NRVM and AMVM cells. And RT-PCR and qPCR was performed as described previously.^38^ Oligonucleotide sequences of primers specific for TRPV4 were TRPV4: 5′-CCCGAGAGAACACCAAGTTTG-3′ (forward), and 5′-GACCGTCATTGTTAAGCACAGTCT-3′ (reverse), and *β*-ACTIN: 5′-CGTTGACATCCGTAAAGACC-3′ (forward), 5′-TAGAGCCACCAATCCACACA-3′ (reverse). Amplified products were separated on 2% agarose gels in TAE buffer, visualized with 1 *μ*g/ml ethidium bromide. The relative expression quantity 2^-ΔΔCt^ value was calculated to compare the differences among groups. The result for each gene was obtained from at least six independent experiments.

### Western blots

Total protein was extracted from the cultured H9C2, NRVM and AMVM as previous described.^38^ The protein concentrations in the supernatants were measured using a BCA kit (Pierce, Rockford, IL, USA). Protein extracts (20 *μ*g) were run on 10% sodium dodecyl sulfate-polyacrylamide electrophoresis gels, and 10 *μ*l biotinylated protein ladder (Cell Signaling Technology, Danvers, MA, USA, #7727) loaded into a separate lane. Then, it was transferred to a nitrocellulose membrane by Electrophoresis System (Liu-Yi, Beijing, China). After being blocked with 5% nonfat milk for 2 h, the membranes were incubated with the appropriate primary antibodies (Alomone Labs, Jerusalem, Israel, #ACC-034) at 4 °C overnight, followed by incubation with a goat anti-rabbit IgG-HRP secondary antibody (Biossci, Wuhan, China, #BB0820) anti-biotin HRP-linked antibody (Cell Signaling Technology, Danvers, MA, USA, #7075) at a dilution of 1 : 2000. The protein expression levels were visualized using enhanced chemiluminescence method by Bio-Rad ChemiDoc XRS (Bio-Rad, Hercules, CA, USA) and quantified by Image Lab Software. *β*-Actin as an internal reference.

### Intracellular calcium measurement

Intracellular calcium was measured as described previously.^39, 40^ In brief, NRVM and H9C2 cells were loaded with 2 *μ*M Fluo-4/AM (Molecular Probes, Carlsbad, CA, USA, #F14201) for 30 min, washed three times with HEPES buffer that contained (in mM) 130 NaCl, 4.7 KCl, 2.0 CaCl_2_,1.2 MgSO_4_, 1.2 KH_2_PO_4_, 10 HEPES and 11 glucose at pH 7.4. Cells in 96-wells plate were illuminated at 488 nm and fluorescence emissions at 525 nm were captured with the Enspire Multimode Plate Reader (PerkinElmer, Boston, MA, USA). GSK1016790A (100, 300 and 500 nM) was used to induce Ca^2+^ influx. In some experiments, cells were pretreated with 1 *μ*M HC-067047 for 30 min. Changes in [Ca^2+^]_i_ upon response to GSK1016790A were presented as relative changes (F/F0) or fold changes (ΔF/F0), where F is the fluorescence at intermediate Ca^2+^ levels, F0 is the average fluorescence before drug stimulation, ΔF is the mean fluorescence after GSK1016790A stimulation at steady-state minus F0. [Ca^2+^]_i_ was calculated with the formula: [Ca^2+^]_i_ =K_d_ × (F0−F_min_)/(F_max_−F0); where K_d_ is the dissociation constant (345 nM for fluo-4), F_min_ is the fluorescence intensity of the indicator in the absence of Ca^2+^ and is obtained by adding a solution of 5 mM EGTA for 15 min, and F_max_ is the fluorescence of the Ca^2+^ saturated indicator and is obtained by adding a solution of 0.1% Triton-X100 in 2.2 mM CaCl_2_ for 15 min.

### Measurement of LDH, cell viability and apoptosis

Cell death was quantified by analyzing LDH activity with commercial kits (JianCheng Bioengineering Institute, Nanjing, China, #A020-2). Cell viability was measured using the Cell Counting Kit-8 (CCK-8, Dojindo Molecular Technologies, Kyushu, Japan, #CK04) as previously described.^38^ Annexin V and PI fluorescein staining kit (eBioscience, SanDiego, CA, USA, #BMS500FI) were utilized to measure apoptosis by following the manufacturer’s instruction. Apoptosis rate was evaluated by Flow Cytometry (FACS Calibur, BD Biosciences, San Jose, CA, USA).

### Measurement of intracellular ROS generation

ROS production in H9C2 was detected using the Reactive Oxygen Species Assay Kit (Beyotime Institute of Biotechnology, Nantong, China). Briefly, following treatment as mentioned in the section ‘H/R model’ above, the cells were incubated with DCFH-DA (10 *μ*M) at 37 °C for 20 min in the dark. The fluorescence was detected by a fluorescent microscope or a Enspire Multimode Plate Reader (PerkinElmer, Boston, MA, USA) (488 nm excitation and 525 nm emission).

Changes in ROS from myocardium after I/R were detected with a fluorescent indicator dihydroethidium (DHE, Sigma-Aldrich). In brief, transverse croysections (5 *μ*m) of the left ventricle (LV) were incubated with 10 *μ*M DHE for 30 min at 37 °C followed by washing with PBS for three times. Nuclei were counterstained with DAPI for 5 min at room temperature. Images were obtained using an Olympus BX-51 epifluorescence microscope (Olympus, Tokyo, Japan). The numbers of DHE-positive nuclei was counted in five random fields in three nonconsecutive sections per heart at × 400 magnification, and expressed as a percentage of the total number of DAPI-stained nuclei.

### Assessment of mitochondrial membrane potential (Δψm)

Changes of mitochondrial membrane potential (Δψm) were measured by staining with JC-1 (Molecular Probes, Carlsbad, CA, USA, #MP03168). After treatment, H9C2 cells were incubated with 5 *μ*M JC-1 at 37 °C for 30 min. The fluorescence densities of the monomers (green, 485/530 nm) and aggregates (red, 525/590 nm) were detected with a fluorescent microscope or a Enspire Multimode Plate Reader. The ratio of aggregated JC-1 and monomeric JC-1 represented ΔΨm.

### Analysis of mPTP opening with calcein

mPTP opening was assayed by measuring calcein (Molecular Probes, #C3100MP) fluorescence quenched by cobalt chloride (Sigma, #60818), as previously reported. In brief, H9C2 cells were loaded with 2 *μ*M calcein/AM and 2 mM CoCl_2_ at 37 °C for 30 min. After washing, cells were illuminated at 488 nm and an emission wavelength of 525 nm was captured with the Enspire Multimode Plate Reader every 30 s. Results were expressed as a percentage of normoxic group.

### *In vivo* mouse model of I/R and treatment

Adult male C57BL/6 mice were obtained from VITAL RIVER (Beijing, China), bred and maintained on a chow diet in a 12-h light/12-h dark environment at 25 °C in the Tongji Medical School Animal Care. All animal procedures were approved by the Institutional Animal Care and Use Committee, which is certified by Huazhong University of Science and Technology Committee on Animal Care. The surgical procedures of I/R were performed as previously described.^7, 41^ Briefly, male C57BL/6 mice (22–23 g) were anesthetized by intraperitoneal injection of pentobarbital sodium (50 mg/kg), and the heart was exposed through a left thoracotomy at the forth intercostal space. The slipknot was tied around the left anterior descending coronary artery (LAD) 2–3 mm from its origin under a surgical microscope and released after 30 min of ischemia to allow reperfusion. Sham-operated (sham) animals were subjected to the same surgical procedures except that the slipknot was not tied.

The TRPV4 antagonist HC-067047 (10 mg/kg) was intraperitoneally injected beginning at 1 h after reperfusion and then injected every 8 h, and the TRPV4 agonist GSK1016790A (0.025 mg/kg) were administrated via jugular vein on the onset of reperfusion. The concentrations of the above-listed chemicals were selected based on previous reports. HC-067047 were first dissolved in DMSO, and then diluted in 0.9% NaCl solution. GSK1016790A was prepared in 1% DMSO/20% Captisol (sulfobutyl ether-*β*-cyclodextrin, Amresco) and saline. Vehicle mice were injected with the same volume of saline.

### Determination of myocardical infarct size at the end of a 24 -h reperfusion

Mice were briefly re-anesthetized at the end of a 24-h reperfusion, and the LAD was re-ligated and 1 ml of 1% Evans Blue dye was infused into the aorta to delineate the AAR. The LV was isolated and cut into 1-mm-thick transverse slices. In order to differentiate infarcted from viable tissue, slices were incubated in 1% triphenyltetrazolium chloride (TTC, Sigma-Aldrich) in phosphate buffer at pH 7.4 at 37 °C for 10 min, then they were fixed with 10% formaldehyde for 24 h and photos were taken. Regions negative for Evans Blue staining (AAR, red and white) and negative for TTC (IA, white) were calculated by a blinded observer using the computer-assisted planimetry function in ImageJ 6.0 (NIH, Bethesda, MD, USA). The myocardial infarct size was expressed as a percentage of IA over total AAR. Serum troponin T levels were evaluated as a biomarker for cardiac damage using a quantitative assay (Roche Diagnostics GmbH, Mannheim, Germany) as previously described.

### Echocardiographic analysis of cardiac function

A Vevo 2100 high-resolution microimaging system with a 30 MHz transducer was used (Visualsonic, Toronto, Ontario, Canada). Mice were anesthetized with 1.5% isoflurane and two-dimensional echocardiographic views of the mid-ventricular short axis and parasternal long axes were obtained. M-mode images were used to measure LV and LV EF and FS, which were acquired by a technician who was blinded to the treatment groups. Data analysis was performed using the Visualsonics data analysis suite.

### Statistical analysis

All values are expressed as mean±S.E.M., and were analyzed for at least six independent experiments. Two-tailed *t*-tests or one-way ANOVA followed by Bonferroni’s post-hoc test were performed to analyze differences with group comparison. Values of *P*<0.05 were considered statistically significant.
