# Supplementary material for: Activation of transient receptor potential vanilloid 4 involves in hypoxia/reoxygenation injury in cardiomyocytes
Source: Cell Death Dis. 2017 May 25;8(5):e2828–. doi: 10.1038/cddis.2017.227 (PMC5520739; doi:10.1038/cddis.2017.227)
Supplement: Supplementary Figure Legend [file cddis2017227x2.docx]

**Figure S1. Effects of TRPV4 antagonist HC-067047 and TRPV4 agonist GSK1016790A on ∆Ψm and mPTP opening levels in H9C2 on under normoxia.** Representative images (A) and quantitative analysis (B) of ∆Ψm level was assessed by the lipophilic cationic probe JC-1 using fluorescence microscopy and a Enspire multimode plate reader, respectively. Scar bar: 50 μm (C) mPTP opening was measured with the ratio of calcein fluorescences density using a Enspire multimode plate reader. The results are represented as mean ± SEM from≥6 independent experiments.
